# Physical Investigations of (Co, Mn) Co-Doped ZnO Nanocrystalline Films

**DOI:** 10.3390/nano10081507

**Published:** 2020-07-31

**Authors:** Bechir Yahmadi, Olfa Kamoun, Badriyah Alhalaili, Safia Alleg, Ruxandra Vidu, Najoua Kamoun Turki

**Affiliations:** 1 Physics Department, Common First Year dean ship, Umm Al-Qura University, Makkah 21955, Saudi Arabia; bmyahmadi@uqu.edu.sa; 2 Physics Laboratory of Condensed Matter, Faculty of Sciences of Tunis, Tunis El Manar University, Tunis 2092, Tunisia; n.najouakamoun@gmail.com; 3 Physics of Semiconductor Devices Unit, Faculty of Sciences of Tunis, Tunis El Manar University, Tunis 2092, Tunisia; 4 Nanotechnology and Advanced Materials Program, Kuwait Institute for Scientific Research, Safat 13109, Kuwait; bhalaili@kisr.edu.kw; 5 Laboratoire de Magnétisme et de Spectroscopie des Solides LMSS, Université de Badji Mokhtar, Sidi Amar, Annaba 23220, Algerie; safia.alleg@univ-annaba.dz; 6 Department of Electrical and Computer Engineering, University of California, Davis, CA 95616, USA

**Keywords:** diluted magnetic semiconductor, nanocrystallites, optical properties, XRD, SEM, room temperature ferromagnetism

## Abstract

Undoped as well as (Co, Mn) co-doped Zinc oxides have been effectively developed on glass substrates, taking advantage of the spray pyrolysis procedure. The X-ray diffraction XRD as well as X-ray photoelectron spectroscopy (XPS) measurements have recognized a pure hexagonal wurtzite form of ZnO, and no other collateral phases such as MnO_2_ or CoO_2_ have been observed as a result of doping. The calculated values of the texture coefficient (TC) were between 0.15 and 5.14, indicating a dominant orientation along the (002) plane. The crystallite size (D) varies with the (Co, Mn) contents. The dislocation density (δ) as well as the residual microstrains increased after Co and Mn doping. Furthermore, the surface morphology of the films has been affected significantly by the Co and Mn incorporation, as shown by the scanning electron microscopy (SEM) investigation. The study of the optical properties exhibits a red shift of the band gap energy (E_g_) with the (Co, Mn) co-doping. The magnetic measurements have shown that the undoped and (Co, Mn) co-doped ZnO thin films displayed room-temperature ferromagnetism (RTFM).

## 1. Introduction

Over the past few years, there has been a growing interest in ZnO, which is recognized as a diluted magnetic semiconductor (DMS) [1,2,3,4]. A diluted magnetic semiconductor can be achieved through the incorporation of transition-metal (TM) elements including Ni, Cr, Co, Fe, and Mn into a non-magnetic semiconductor. DMS materials have inspired a great deal of academic and industrial research regarding their great potential in several fields, such as optoelectronics, sensors, light emitting diodes (LED), nanoelectronics, photonics, and spintronic devices [5,6,7,8,9,10,11,12,13]. Metal oxide semiconductors such as TiO_2_, SnO_2_, In_2_O_3_, and ZnO are known to exhibit DMS behavior as a result of TM-doping [14,15,16,17,18,19,20,21,22,23]. Thanks to its fascinating features, such as large energy bandgap E_g_ (≈3.37 eV), relatively great exciton binding energy (≈60 meV), high electrical conductivity, as well as high temperature magnetic response [24,25,26], ZnO material is a II-VI binary semiconducting material and is considered to be one of the best candidates for the modern electronic industry. ZnO crystallizes in three main crystallographic varieties—namely, wurtzite, zinc blend, and rock salt. However, at room temperature the hexagonal wurtzite form is the most stable one [27].

Several techniques have been used to prepare TM-doped ZnO compounds. For instance, Zn_0.8_Co_0.2_O and Zn_0.8_Ni_0.1_Co_0.1_O nanoparticles have been elaborated by Vijayaprasath et al. [26] using the chemical co-precipitation method. According to their magnetic measurements, induced ferromagnetism has been observed either in Co or Ni-doped ZnO, while a weak ferromagnetic behavior was noticed as a result of the co-doping of Co with Ni/ZnO nanoparticles. Khan et al. [28] have synthesized pure and (Co, Mn) co-doped ZnO nanoparticles by the chemical precipitation technique. Based on the X-ray diffraction (XRD) analysis, there was no change in the wurtzite-type structure of their synthesized ZnO samples with doping, whereas there is a decrease in the average nanoparticle size after doping ZnO with (Co, Mn). On the other hand, a significant transition from the diamagnetic state for pure ZnO nanoparticles to an obvious ferromagnetic state for Zn_0.96−*x*_Mn_0.04_Co*_x_*O (where *x* ranged from 0 to 4 wt.%) was reported [28]. The highest magnetization was observed for 1 wt.%—e.g., Zn_0.95_Mn_0.04_Co_0.01_O—with a remnant magnetization (Mr) of about 0.25 × 10^−2^ emu/g. The study of the magnetic properties of ZnO material prepared by the sol gel auto-combustion method revealed the absence of ferromagnetism behavior in these samples despite the Mn^2+^ doping, as reported by Birajdar et al. [29]. However, they exhibit a paramagnetic nature at room temperature. The optical band gap of ZnO nanopowders elaborated by the co-precipitation method has been found to be significantly affected by Al and (Al, Mn) doping, as reported by Belkhaoui et al. [30]. In their previous investigation, Kamoun et al. [31] explored the effect of Eu doping content on certain physical properties of sprayed ZnO compound, where they reported that the lattice parameters were at the maximum values for samples with a ratio of 1% [Eu]/[Zn].

In spite of the extensive studies of the doping and/or co-doping effect on the physical properties of zinc oxide, the room-temperature ferromagnetism (RTFM) effect is still the subject of an extensive debate regarding its origin. The present work aims to examine the impact of the incorporation of Co and Mn atoms on the physical properties of the ZnO compound. For this purpose, ZnO and (Co, Mn) co-doped ZnO nanocrystalline films have been synthesized by the spray pyrolysis technique, which was already described in our previous paper [31]. A comprehensive characterization of the structural, morphological, optical, and magnetic properties of the synthesized (Co, Mn) co-doped ZnO nanocrystalline films was performed.

## 2. Materials and Methods

### 2.1. Undoped and (Co, Mn) Co-Doped ZnO Nanocrystalline Films Synthesis

Pure ZnO nanocrystalline films have been synthesized on glass substrates at 460 °C, under the conditions described previously by Kamoun et al. [31]. Briefly, ZnO nanoparticles were obtained from a mixture of zinc acetate (Zn(CH_3_CO_2_)_2_: 10^−1^ M) and propanol. The pH of the precursor mixture was adjusted to 5 by adding acetic acid. The carrier gas used was nitrogen (pressure ≈ 0.35 bar), which was purged through a 0.5 mm diameter nozzle. The nozzle-to-substrate plane distance was fixed at the optimal value of 27 cm, as demonstrated by K. Boubaker et al. [32], and the spray solution flow rate was 4 mL/min. After deposition, the ZnO films were cooled down to room temperature. Similarly, (Co, Mn) co-doped ZnO nanocrystalline films have been prepared by adding cobalt sulphate heptahydrate and manganese chloride tetrahydrate (CoSO_4_·7H_2_O, ≥ 99% and MnCl_2_·4H_2_O, ≥ 98%, Sigma-Aldrich (St. Louis, MO, USA) as Co and Mn cations precursors to the host precursor solution. The atomic ratios of the (Co, Mn) dopants were (0%, 0%), (1%, 1%), (2%, 1%), (1%, 2%), and (2%, 2%).

### 2.2. Characterization Techniques

The crystallographic structure characteristics of the undoped and (Co, Mn) co-doped ZnO samples were performed employing an X-ray diffractometer equipment type Philips PW 1729, equipped with a monochromatic radiation source (Cukα, *λ* = 1.54056 Å, Philips PW 1729 system, Cambridge, MA, USA). The X-ray photoelectron spectroscopy (XPS) analyses were carried out using a Thermo-VG Scientific MultiLab, ESCA Probe with Al Kα 1486.7 eV (Fisher Scientific, Waltham, MA, USA) as an exciting source in order to identify the chemical elements and their oxidation states.

In addition, the surface morphology of the thin films has been explored by electronic scanning microscopy (class EDAX XL 30 (S.E), EDAX, Mahwah, NJ, USA). The optical properties of all the ZnO samples, in terms of the transmission (T%) and optical band gap energy (E_g_), were analyzed in the 250–2500 nm range with a spectrophotometer device (type Perkin Elmer Lambda 950). The magnetic properties studies were accomplished using a vibrating sample magnetometer (VSM, Microsense EV9, DMG MORI Manufacturing USA, Inc., Davis, CA, USA). The magnetization (M) measurements were conducted at room temperature in the field range from −20 kOe to 20 kOe.

## 3. Results and Discussion

### 3.1. Crystallographic Structure

**Figure 1:**
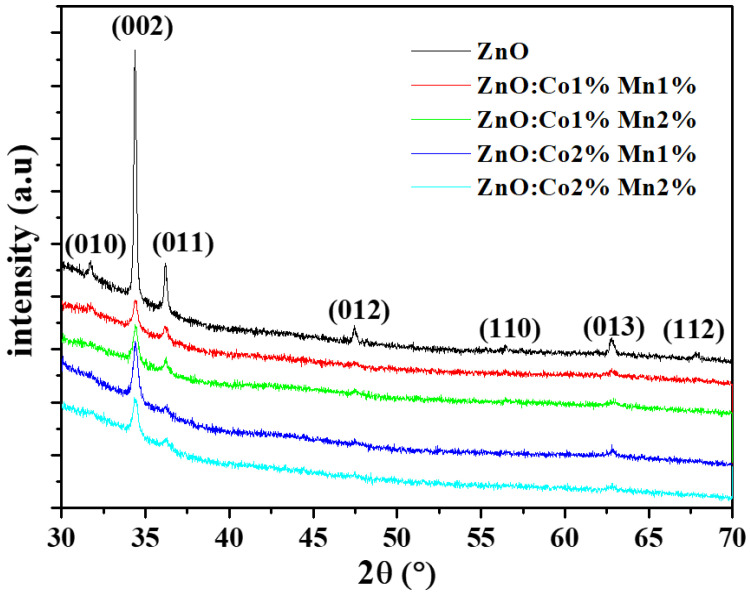
X-ray diffraction XRD spectra of the undoped and (Co, Mn) co-doped ZnO samples for different (Co, Mn) contents.

The XRD spectra of the undoped and (Co, Mn) ZnO samples are outlined in Figure 1, which clearly demonstrates the main peaks for ZnO, such as (010), (002), (011), (012), (110), (013), and (112) orientations. According to the JCPDS Card n°: 036-1451, these orientations are assigned to the hexagonal wurtzite structure of ZnO material. In addition, all the samples show *c*-axis-oriented growth (002). The incorporation of (Co, Mn) seems to be without noticeable impact on the crystal structure of the ZnO samples. Indeed, no change in the preferential orientation and no secondary phases such as MnO_2_ or CoO_2_ in the ZnO host material were observed as a result of doping. The same behavior was reported by other research groups [3,5,33,34,35]. Besides this, it is worth mentioning that some of these diffraction peaks become weaker or disappear as the (Co, Mn) content changes. This change in intensity is accompanied by a very small shift towards larger angles in the broad peaks of (Co, Mn) co-doped ZnO nanocrystalline films with reference to the undoped ones. The shift in the position of the peak to higher angles as a result of (Co, Mn) co-doping may be explained by the contraction of the lattice parameters due to the size difference between the Zn atoms and the dopants. Moreover, since there is no secondary phases detected in the (Co, Mn) co-doped ZnO nanocrystalline films, the Co^2+^ and Mn^2+^ dopants have either replaced the Zn^2+^ or integrated inside the interstitial sites with no noticeable alteration in the ZnO host structure [36].

The mean grain size (D) of the deposited nanocrystalline films has been estimated by the Rietveld method [37]. The evaluated grain size (D) values are recorded in Table 1. On the basis of these results, it is evident that there is a significant impact of the (Co, Mn) content on the (D) value. D is equal to 104.0 nm for the undoped ZnO sample and 26.4–43.0 nm for the (Co, Mn) co-doped ZnO samples. The highest value of 43.0 nm was obtained for the (Co 1%, Mn 2%) co-doped ZnO. Although our results differ to some extent from those of Poornaprakashet et al. [7] and Sundaramet et al. [3], they have a number of similarities with those reported by Abdullahi et al. [25] and Voicu et al. [35]. The decrease in the grain size with the (Co, Mn) incorporation may be due to the distortion generated by the foreign impurities in the host ZnO lattice, as well as by a reduction in the nucleation rate along with a decrease in the growth rate of ZnO nanocrystalline films induced by the presence of the Co^2+^ and Mn^2+^ dopants.

The experimental values of the crystal parameters *a* and *c* are obtained by the Rietveld refinement for undoped and (Co, Mn) co-doped ZnO nanocrystalline films (Table 1). The ratio *c*/*a* is equal to 1.60 for all the samples except for (Co 2%, Mn 2%), where the *c*/*a* is 1.59. In addition, the lattice strains A and C along the *a*-axis and *c*-axis, respectively, were estimated using the following formulae:

A = (*a* − *a*_0_)/*a*_0,_ (1)

As well as:

C = (*c* − *c*_0_)/*c*_0._ (2)

The values calculated with Equations (1) and (2) are reported in Table 1, where one can see that A and C for ZnO are equal to 0.46% and 0.51%, respectively. The variations in A and C with the amount of co-doping can also be observed. The negative values of A and C may be explained by the compression of crystal parameters induced by co-doping ZnO, especially for (Co, Mn) equal to (Co 2%, Mn 2%), for which there is a small compression along the *c*-axis; for (Co 1%, Mn 1%), there is a small compression of both the *a* and *c* axis, while for other the (Co, Mn) content the crystal seems to have a small expansion (Table 1). The rate of micro deformations (σ^2^)^1/2^ is equal to 10^−4^% for ZnO, while it varies in the range of (10^−5^–2.10^−3^) for all the (Co, Mn) co-doped ZnO samples. The occupancy coefficients are (Zn:1—O:1) for ZnO, (Co 1%, Mn 1%), and (Co 1%, Mn 2%) co-doped ZnO, but this value becomes (Zn:0.9—O:1) and (Zn:0.87—O:1) for (Co 2%, Mn 1%) and (Co 2%, Mn 2%) co-doped ZnO, respectively.

Furthermore, additional insights on the impact of the Co and Mn atoms incorporation on the structural properties of the sprayed ZnO material can be obtained from the texture coefficient (TC), the dislocation density (δ), and the internal strain (ε). The value of the texture coefficient (TC), which indicates the abundance of grains in a (hkl) orientation, was calculated using the Harris method as follows [38,39]:

TC (hkl) = [I_hhk_/I_0_] ⁄ [*n*^−1^ × (∑I_hkl_)⁄I_0_)], (3)

where *n*, I_hkl_, and I_0_ are the number and the intensity of reflections, respectively. The measured intensity of the (hkl) orientation and the corresponding intensity of the XRD reference are provided by the JCPDS 036-1451 card. The results are summarized in Table 2. The TC value varies from 0.15 to 5.14 for all the samples, indicating a dominant orientation along the (002) plane. As the (Co:Mn) content varies, the TC value changes randomly. This result could reveal that the insertion of (Co, Mn) elements may deteriorate the crystallinity of the growing ZnO films owing to the ionic radii differences between the Zn, Co, and Mn atoms [28]. In addition, the (Co, Mn) doping causes a reduction in the intensity and widening of diffraction peaks, which means that smaller crystallites develop in the doped ZnO compared to the undoped ZnO as a consequence of the induced lattice disorder.

The amount of defects representing the displacement of the crystal structure when additional impurities are incorporated into a crystal can be evaluated by calculating the dislocation density (δ). The dislocation density, δ, defined as the number of intercepted positions by the dislocation lines per unit area in the plane perpendicular to the dislocation lines (lines/m^2^) [40], was estimated using the Williamson and Smallman formula [40,41,42,43,44,45], as given by the following equation:

δ = 1⁄*D*^2^, (4)

where *D* is the crystallite size of the ZnO samples. The calculated values of dislocation density are summarized in Table 3. 

The dislocation density is equal to 0.92 × 10^14^ lines/m^2^ for the undoped ZnO (0%, 0%) film. The dislocation density increases from 5.4 to 14.3 × 10^14^ lines/m^2^ as the dopant content increases. These results emphasize that there is an increase in lattice imperfections in the (Co, Mn) co-doped ZnO.

The internal strain (ε) of ZnO and (Co%, Mn%) co-doped ZnO nanocrystalline films has been estimated using the following relationship [3]:

ε = β × cosθ⁄4 = 0.96*λ*/4D. (5)

The corresponding calculated values of (ε) are recapped in Table 3. Compared to the undoped ZnO nanocrystalline films, the strain value seems to be increased with doping. It equals 0.59 × 10^−2^ for pure ZnO, whereas it ranges from 1.61 × 10^−2^ to 1.79 × 10^−2^ for (Co%, Mn%) co-doped ZnO. This behavior is due to the incorporation of the doping elements Co and Mn in the ZnO unit cells, which generates alteration either in the size and/or of the shape of the synthesized ZnO nanocrystalline films [26], accompanied by the creation nearby of the Co^2+^ and Mn^2+^ ions of charge imbalance as well as the development of some crystallographic defects.

### 3.2. X-ray Photoelectron Spectroscopy Analysis

**Figure 2:**
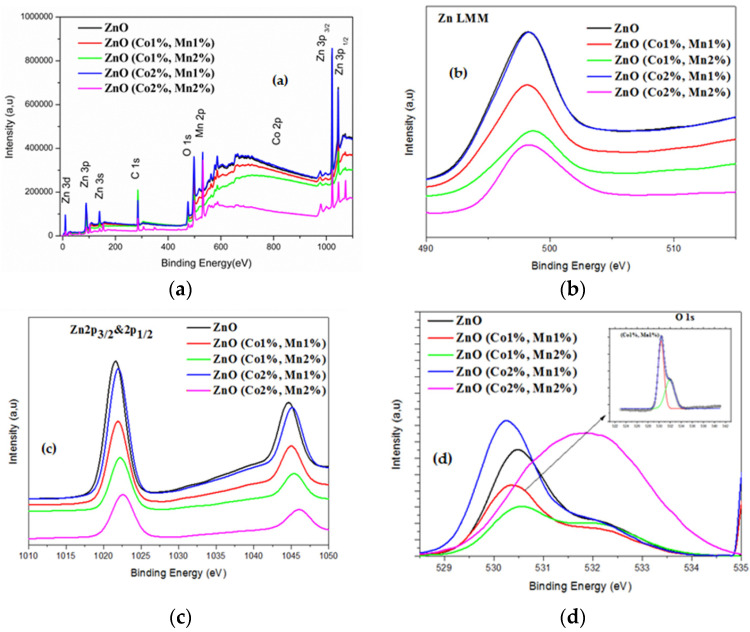
X-ray photoelectron spectroscopy (XPS) spectra of (**a**) the ZnO and (Co, Mn) co-doped ZnO; (**b**) the Zn LMM regions and (**c**) the Zn 2p3/2 and 2p1/2 regions; (**d**) the O 1s region of ZnO and (Co, Mn) co-doped ZnO (note: the XPS spectra were recorded at room temperature).

X-ray photoelectron spectroscopy (XPS) is a quantitative technique that offers further details about the incorporation of the Co and Mn dopants into the host lattice of ZnO. Figure 2 illustrates the full XPS spectra of all the ZnO samples, as well as the high resolution XPS spectra of Zn and O.

In the XPS spectra, the standardization of the peak positions was conducted according to the C1s peak at 284.6 eV. In Figure 2a, four peaks corresponding to the Zn, O, Co, and Mn elements (in the case of (Co, Mn), co-doped samples) can be identified, which confirm the existence of Co and Mn dopants in the ZnO host material. No other extra peaks have been detected, which also demonstrate the single-phase structure of the ZnO synthesized in our work. To further investigate the oxidation states of the Zn, O, Co, and Mn elements, a refined scan closely to each peak has been carried out for the ZnO and (Co, Mn) co-doped ZnO samples. The Zn LMM Auger spectra (Figure 2b) exhibit only one peak, localized at a binding energy ranging from 497.8 to 498.2 eV (Table 4), which most likely originated from the interstitial Zinc (Zn_i_) as well as Zn-O bonds.

The high-resolution XPS spectra (not shown) of the Co and Mn peaks for all the co-doped samples has shown only one broad peak centered around 781.1 and 640.4 eV (Table 4), in agreement with the binding energies of Co 2p3/2 and Mn 2p3/2, respectively. Figure 2c shows two marked peaks localized at 1021.6 and 1044.6 eV, in accordance with the binding energies of Zn 2p3/2 and Zn 2p1/2, respectively, for ZnO [46]. As the atomic ratios Co(%) = [Co]/[Zn] and Mn(%) = [Mn]/[Zn] change, the binding energy of Zn 2p3/2 increases from 1021.6 for ZnO to 1022.6 for the (Co 2%, Mn 2%) co-doped ZnO sample. In addition, the spin-orbital splitting energy (∆E) is around 23.1 eV, revealing that Zn is present as the Zn^2+^ state regardless the Co and Mn doping level used. The weakness of the peak intensities of the Co 2p and Mn 2p XPS spectra probably owe to the low level of Mn and Co doping. The high-resolution XPS spectra analysis of the oxygen element and the deconvolution of the associated asymmetric peak (O 1s) of the undoped ZnO sample have shown two Gaussian signals with low and high binding energies. The first one, centered at about 530.5 eV, could be assigned to O^2−^ ions tied to Zn^2+^ in the ZnO lattice. The peak of the high binding energy localized at 531.8 eV can most likely be assigned to oxygen vacancies [47].

### 3.3. Surface Morphology

**Figure 3:**
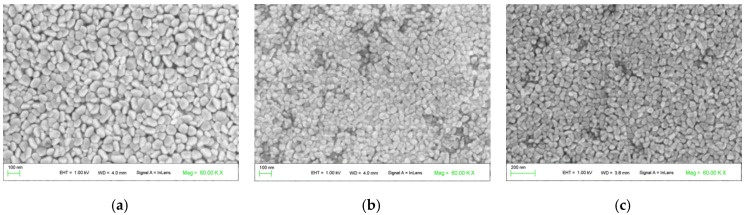
SEM (scanning electron microscopy) images of the surface morphology of the ZnO and (Co, Mn)-co-doped ZnO samples: (**a**) ZnO; (**b**) (Co 1%, Mn 1%) co-doped ZnO; (**c**) (Co 2%, Mn 1%) co-doped ZnO.

The scanning electron micrographs of three samples corresponding to ZnO, (Co 1%, Mn 1%) co-doped ZnO, and (Co 2%, Mn 1%) co-doped ZnO are presented in Figure 3. For ZnO, a uniform grain size distribution was observed (Figure 3a), with a mean grain size of about 65 nm. As (Co, Mn) is added to ZnO, the surface morphology becomes less uniform, which is associated with a decrease in the corresponding crystallite size (Figure 3b,c). The change is the surface morphology may be explained by the differences in the ionic radius of Zn^2+^, Co^2+^, and Mn^2+^, which are approximately equal to 0.60, 0.65, and 0.80 Å, respectively [28]. Thus, it would appear that the incorporation of (Co, Mn) transition metal increases the disorder in the ZnO thin film, which also affected its crystallinity.

### 3.4. Optical Investigations

**Figure 4:**
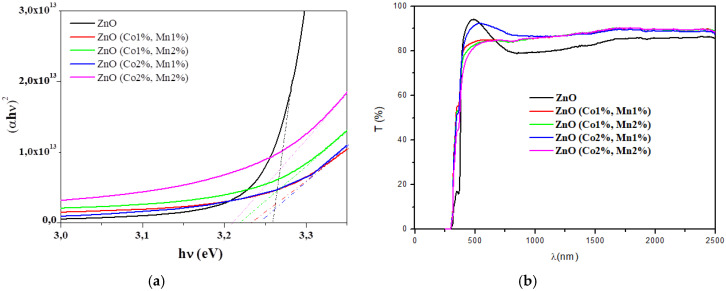
(**a**) Transmittance (T) spectra; (**b**) (αhν)^2^ vs. the incident photon energy (hν) of the ZnO and (Co, Mn) co-doped ZnO samples.

The transmittance spectra of the ZnO and (Co, Mn) co-doped ZnO samples are shown in Figure 4a. The (Co 2%, Mn 1%) co-doped ZnO film is more transparent than ZnO in the visible range. In the near infra-red region, the transmittances of the (Co, Mn) co-doped ZnO samples are greater than those of ZnO.

The fundamental absorption edge (E_g_) of the films is in the range of 250–400 nm and can be attributed to a direct transition, where the absorption coefficient, α, can be correlated to the optical bandgap E_g_, as given by [48,49,50]:

αhν = K(hν − E_g_)^½^, (6)

where hν and K represent the photon energy and a constant of proportionality, respectively.

The minor absorption observed in the intrinsic front absorption edge in the wavelength range of 290–390 nm may be due to the Urbach tails caused by the presence of the states in the band gap near to the bottom of the conduction band and/or to the maximum of the valence band, which may be due to the lattice defects.

The extrapolation to the energy axis of the straight line zone of (αhν) ^2^ against (hν) graphs gives the E_g_ values for each sample (Figure 4b, Table 5). The optical bandgap slightly decreases with co-doping contents. In fact, the optical bandgap E_g_ of 3.25 eV for the ZnO sample decreases to 3.21 eV for the (Co 2%, Mn 2%) co-doped ZnO (Table 5). The optical band gap variation may be explained by the fluctuation in the grain size of the corresponding ZnO nanocrystalline films due to the insertion of the Co and Mn dopants. Our results are in agreement with other research groups [31,48,51,52,53].

### 3.5. Magnetic Properties

**Figure 5:**
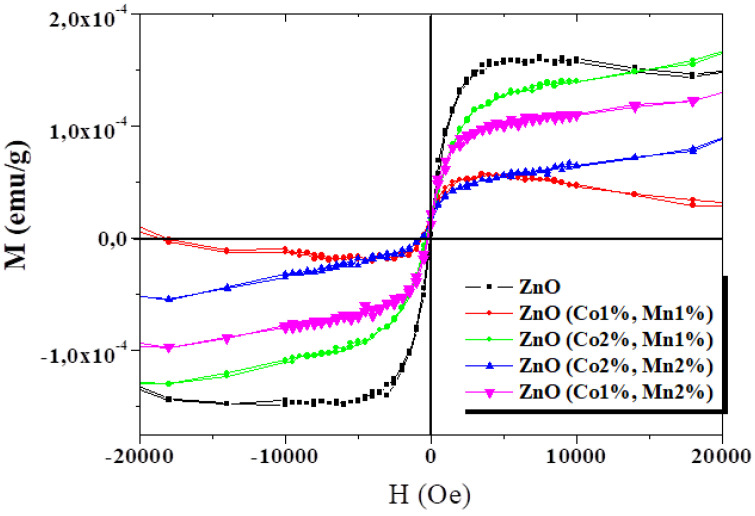
Magnetization as a function of magnetic field at room temperature for the ZnO and (Co, Mn) co-doped ZnO samples.

The magnetization (M) measurements were performed at room temperature as a function of the applied magnetic field (H) for ZnO and (Co, Mn) co-doped ZnO (Figure 5). From this figure showing the M–H curves for all the samples, an explicit ferromagnetic behavior at room temperature can be deduced. Although the presence of ferromagnetism at room temperature in certain oxide semiconductor thin films, nanorods, nanowires, and nanoparticles has been reported in the literature [54,55,56,57], there is no information about (Co, Mn) co-doped ZnO grown by spray pyrolysis. 

A visible decrease in magnetization was observed for (Co1%, Mn1%) ZnO, which has a similar M-H variation to that of ZnO films. When the atomic ratio of Co or Mn dopants increases, the shape of the M–H curve changes. The magnetization of the (Co, Mn) co-doped ZnO thin films tend to increase as the applied magnetic field increases, with the non-expected saturation of the magnetization values. This behavior is comparable to those reported on the (Co, Ni), (Co, Ga), and (Co, Mn) co-doped ZnO nanoparticles synthesized via the co-precipitation route by Vijayaprasath et al. [58], Lu et al. [2], and Khan et al. [28], respectively.

Moreover, several other researchers reported on the absence of room temperature ferromagnetism for Co-doped [59] or (Co, Mn) co-doped zinc oxide [60,61]. However, another significant trend recognized as “d_0_ ferromagnetism” has been observed by other research groups in some un-doped semiconducting oxides [62,63,64]. The RTFM effect observed in metal oxide compounds is still a subject of extensive debate regarding its origin, and it may strongly depend on the processing parameters, such as the synthesis method, synthesis temperature, grain size, annealing treatment, substrate type, film thickness, and most likely on the doping/co-doping process, as well as on dopant natures. Khan et al. [28] suggested that the RTFM observed in the corresponding co-doped ZnO nanocrystalline films could result from either extrinsic or intrinsic magnetism sources. Gacic et al. [65] believe that magnetism results from the formation of certain local clusters or from some secondary phases generated in the host matrix, but not by the transition metal atoms used as dopants. Chen et al. [64] reported that oxygen-related vacancies may play an important role in the RTFM detected in ZnO pellets. In our samples, the RTFM probably originates from the structural defects generated in ZnO samples grown by spray pyrolysis.

## 4. Conclusions

Undoped and (Co, Mn) co-doped ZnO nanocrystalline films with various Co and Mn contents were successfully synthesized on glass substrate by spray pyrolysis. The crystallographic structure, surface morphology and optical and magnetic characterization of the films were studied. The XRD study revealed the development of the (002) preferential direction according to the hexagonal wurtzite structure of ZnO, and no collateral phases such as MnO_2_ or CoO_2_ have been observed as evinced by the XRD and XPS studies. It has been shown that the crystallite size (D), dislocation density (δ), and residual microstrain (ε) parameters are significantly affected by Co and Mn incorporations in the ZnO material. The SEM has shown that the morphology and size of the nanocrystalline grains were affected by the amounts of (Co, Mn) dopants. The optical investigations have shown a relatively high optical transmittance, with a slight blue shift in the band gap energy (E_g_) with respect to the Co and Mn contents. The E_g_ varies between 3.25 and 3.21 eV, respectively, for the ZnO and (Co 2%, Mn 2%) co-doped ZnO samples. This result suggested that the highest feature of the sprayed ZnO nanocrystalline films can be tuned by varying the Co and Mn content in the ZnO lattice. Magnetic measurements have shown that the undoped and (Co, Mn) co-doped ZnO thin films exhibited room temperature ferromagnetism. This RTFM in our samples may originate from the spray pyrolysis technique growth. The present investigation not only shows a room temperature *d*_0_ ferromagnetism in pure ZnO thin films, but also demonstrates a fine-tunable RTFM in (Co, Mn) co-doped ZnO diluted magnetic semiconductor (DMS) ZnO material, which is of great interest in the field of spintronic devices.

## Figures and Tables

**Table 1:** Mean grain size values (D), perfect crystal parameters (*a*_0_, *c*_0_) of ZnO and (*a*, *c*) of (Co%, Mn%) co-doped ZnO samples, and (σ^2^)^1/2^ is the rate of micro deformation.

ZnO (Co%:Mn%)					
Structural Parameters	0%:0%	1%:1%	1%:2%	2%:1%	2%:2%
D (nm) ± 0.1	104.0	30.0	43.0	34.0	26.4
a(nm) ± 10^−4^	0.3256	0.3255	0.3254	0.3256	0.3253
c(nm) ± 10^−4^	0.5214	0.5211	0.5210	0.5213	0.5214
*c/a*	1.60	1.60	1.60	1.60	1.59
A(%) = (*a* − *a*_0_)/a_0_	0.46	−0.43	0.43	0.43	0.21
C(%) = (*c* − *c*_0_)/c_0_	0.51	−0.44	0.44	0.44	−0.09
⟨σ^2^⟩^1/2^ (%)	10^−4^	10^−4^	2.10^−3^	10^−5^	10^−4^
Occupancy	Zn:1 O:1	Zn:1 O:1	Zn:1 O:1	Zn:0.9 O:1	Zn:0.87 O:1

**Table 2:** Values of the texture coefficient (TC) of undoped and (Co%, Mn%) co-doped ZnO nanocrystalline films.

TC					
(hkl)	0% 0%	1% 1%	1% 2%	2% 1%	2% 2%
**(010)**	0.2	0.44	---	---	---
**(002)**	5.14	2.92	2.72	3.14	2.39
**(011)**	0.40	0.55	0.37	0.15	0.39
**(012)**	0.55	0.46	0.54	0.27	---
**(013)**	0.46	0.63	0.37	0.44	0.38

---: Absent peak.

**Table 3:** Internal lattice strain (ε) of undoped and (Co, Mn) co-doped.

ZnO (Co%, Mn%)					
Structural Defects	(0%, 0%)	(1%, 1%)	(1%, 2%)	(2%, 1%)	(2%, 2%)
**δ(10^14^ lines/m^2^)**	0.9	11.1	5.4	8.6	14.3
**ε (10^−2^)**	0.59	1.61	1.79	1.7	1.74

**Table 4:** XPS measurements of binding energy of O 1s, Zn LMM, Zn 2p3/2, Co 2p3/2, and Mn 2p3/2 of the undoped and (Co%, Mn%) co-doped ZnO.

Binding Energy (eV)					
Mn 2p3/2	Co 2p3/2	Zn 2p3/2	Zn LMM	O 1s	(Co%, Mn%)
------	------	1021.6	497.8	530.5	(0%, 0%)
640.4	781.1	1021.9	497.8	530.4	(1%, 1%)
640.2	781.83	1022.1	498.2	530.7	(1%, 2%)
640.4	780.77	1021.9	497.9	530.3	(2%, 1%)
642	780.69	1022.6	498.2	531.8	(2%, 2%)

------: absent peak.

**Table 5:** Band gap energy (E_g_) values of the undoped and (Co, Mn) co-doped ZnO.

ZnO (Co%, Mn%)	(0%, 0%)	(1%, 1%)	(1%, 2%)	(2%, 1%)	(2%, 2%)
**E**_g_ **(eV)**	3.25	3.23	3.22	3.24	3.21

## References

[1] Pan F., Song C., Liu X.J., Yang Y.C., Zeng F. (2008). Ferromagnetism and possible application in spintronics of transition-metal-doped ZnO films. Mater. Sci. Eng..

[2] Lu B., Wang Y., Li W., Zhang W., Ye Y., Zhang L., Ye Z. (2015). Co–Ga codoping effect on preferred growth orientations and properties of ferromagnetic ZnO thin films. J. Magn. Magn. Mater..

[3] Sundaram P.S., Inbanathan S.S.R., Arivazhagan G. (2019). Structural and optical properties of Mn doped ZnO nanoparticles prepared by co-precipitation method. Physica B.

[4] Ahmed S.A. (2017). Structural, optical, and magnetic properties of Mn-doped ZnO samples. Results Phys..

[5] Liu X.C., Chen Z.Z., Zhen B.Z.C., Shi E.W., Liao D.Q. (2010). Structural, optical and electrical properties of Ga-doped and (Ga, Co)-co-doped ZnO films. J. Cryst. Growth.

[6] Tariq M., Li Y., Li W., Yu Z., Li J., Hu Y., Zhu M., Jin H., Li Y., Skotnicova K. (2019). Enhancement of ferromagnetic properties in (Fe, Ni) co-doped ZnO flowers by pulsed magnetic field processing. J. Mater. Sci..

[7] Poornaprakasha B., Chalapathia U., Subramanyamb K., Vattikutic S.V.P., Park S.H. (2020). Wurtzite phase Co-doped ZnO nanorods: Morphological, structural, optical, magnetic, and enhanced photocatalytic characteristics. Ceram. Int..

[8] Chang Y.Q., Wang D.B., Luo X.H., Xu X.Y., Chen X.H., Li L., Chen C.P., Wang R.M., Xu J., Yu D.P. (2003). Synthesis, optical, and magnetic properties of diluted magnetic semiconductor Zn_1– x_Mn_x_O nanowires via vapor phase growth. Appl. Phys. Lett..

[9] Schneider C.M. (2020). Spintronics: Surface and interface aspects. Surf. Interface Sci..

[10] Wang Q., Sun Q., Jena P. (2007). Ab initio study of electronic and magnetic properties of the C-co-doped Ga_1–*x*_Mn*_x_*N (1010) surface. Phys. Rev. B.

[11] Klingshirn C. (1975). Optical properties of bound and localized excitons and of defect states. Phys. Status Solidi B.

[12] Xu X.Y., Cao C.B. (2009). Structure and ferromagnetic properties of Co-doped ZnO powders. J. Magn. Magn. Mater..

[13] Dietl T. (2010). A ten-year perspective on dilute magnetic semiconductors and oxides. Nat. Mater..

[14] Sato K., Yoshida H.K. (2000). Material design for transparent ferromagnets with ZnO-based magnetic semiconductors. J. Appl. Phys..

[15] Lin Y., Jiang D., Lin F., Shi W., Xueming M. (2007). Fe-doped ZnO magnetic semiconductor by mechanical alloying. J. Alloys Compd..

[16] Sharma P.K., Dutta R.K., Pandey A.C., Layek S., Verma H.C. (2009). Effect of iron doping concentration on magnetic properties of ZnO nanoparticles. J. Magn. Magn. Mater..

[17] Bae S.Y., Na C.W., Kang J.H., Park J. (2005). Comparative structure and optical properties of Ga-, In-, and Sn-doped ZnO nanowires synthesized via thermal evaporation. J. Phys. Chem. B.

[18] Wen J.G., Lao J.Y., Wang D.Z., Kyaw T.M., Foo Y.L., Ren Z.F. (2003). Aberration-corrected transmission electron microscopy for advanced materials characterization. Chem. Phys. Lett..

[19] Wolf S.A., Awschalom D.D., Buhrman R.A., Daughton J.M., Svon M., Roukes M.L., Chtchelkanova A.Y., Treger D.M. (2001). Spintronics: A spin-based electronics vision for the future. Science.

[20] Fert A. (2008). Origin, development, and future of spintronics (Nobel lecture). Angew. Chem. Int. Ed..

[21] Dietl T., Ohno H., Matsukura F., Cibert J., Ferrand D. (2000). Zener model description of ferromagnetism in Zinc-blende magnetic semiconductors. Science.

[22] Ahn G.Y., Park S.I., Kim C.S. (2006). Enhanced ferromagnetic properties of diluted Fe doped ZnO with hydrogen treatment. J. Magn. Magn. Mater..

[23] Sun S., Wu P., Xing P. (2012). d0 ferromagnetism in undoped n and p-type In_2_O_3_ films. Appl. Phys. Lett..

[24] Khan R., Fashu S., Rehman Z.U. (2017). Structural, dielectric and magnetic properties of (Al, Ni) co-doped ZnO nanoparticles. J. Mater. Sci..

[25] Abdullahi S.S., Köseoğlu Y., Güner S., Kazan S., Kocaman B., Ndikilar C.E. (2015). Synthesis and characterization of Mn and Co co-doped ZnO nanoparticles. Superlattices Microstruct..

[26] Vijayaprasath G., Murugan R., Asaithambi S., AnandhaBabu G., Sakthivel P., Mahalingam T., Hayakawa Y., Rav G. (2016). Structural characterization and magnetic properties of Co co-doped Ni/ZnO nanoparticles. Appl. Phys. A.

[27] Srinivasulu T., Saritha K., Reddy K.T.R. (2017). Synthesis and characterization of Fe-doped ZnO thin films deposited by chemical spray pyrolysis. Mod. Electron. Mater..

[28] Khan R., Zulfiqar Z., Fashu S., Ur Rehman Z., Khan A., Ur Rahman M. (2018). Structure and magnetic properties of (Co, Mn) co-doped ZnO diluted magnetic semiconductor nanoparticles. J. Mater. Sci..

[29] Birajdar S.D., Alange R.C., More S.D., Murumkar V.D., Jadhav K.M. (2018). Sol-gel auto combustion synthesis, structural and magnetic properties of Mn doped ZnO nanoparticles. Procedia Manuf..

[30] Belkhaoui C., Mzabi N., Smaoui H., Daniel P. (2019). Enhancing the structural, optical and electrical properties of ZnO nanopowders through (Al + Mn) doping. Results Phys..

[31] Kamoun O., Boukhachem A., Yumak A., Petkova P., Boubaker K., Amlouk M. (2016). Europium incorporation dynamics and some physical investigations within ZnO sprayed thin films. Mater. Sci. Semicond Process..

[32] Boubaker K., Chaouachi A., Amlouk M., Bouzouita H. (2007). Enhancement of pyrolysis spray disposal performance using thermal time-response to precursor uniform deposition. Eur. Phys. J. Appl. Phys..

[33] Li G.R., Qu D.L., Zhao W.X., Tong Y.X. (2007). Electrochemical deposition of (Mn,Co)-co-doped ZnO nanorods arrays without any template. Electrochem. Commun..

[34] Liu H., Li W., Zhang X., Sun Y., Song J., Yangn J., Gao M., Liu X. (2015). Comparative study of room temperature ferromagnetism in Cu, Co co-doped ZnO film enhanced by hybridization. Ceram. Int..

[35] Voicu G., Miu D., Ghitulica C.D., Jinga S.I., Nicoara A.I., Busuioc C., Holban A.M. (2020). Co doped ZnO thin films deposited by spin coating as antibacterial coating for metallic implants. Ceram. Int..

[36] Mallikan A.N., Reddy A.R., Sowribabu K., Reddy K.V. (2015). Structural and optical characterization of Zn_0.95_×Mg_0.05_Al×O nanoparticles. Ceram. Int..

[37] Wiles D.B., Young R.A. (1981). A new computer program for Rietveld analysis of X-ray powder diffraction patterns. J. Appl. Cryst..

[38] Snega S., Ravichandran K., Baneto M., Vijayakumar S. (2015). Simultaneous enhancement of transparent and antibacterial properties of ZnO films by suitable F doping. J. Mater. Sci. Technol..

[39] Mrabet C., Kamoun O., Boukhachem A., Amlouk M., Manoubi T. (2015). Some physical investigations on hexagonal-shaped nanorods of lanthanum-doped ZnO. J. Alloys Compd..

[40] Barabash R.I., Ice G.I. (2014). Diffraction analysis of defects: state of the art. Strain and Dislocation Gradients from Diffraction. Spatially-Resolved Local Structure and Defects.

[41] Williamson G.K., Smallman R.E. (1956). III. Dislocation densities in some annealed and cold-worked metals from measurements on the X-ray debye-scherrer spectrum. Philos. Mag..

[42] Yahmadi B., Kamoun N., Guasch C., Bennaceur R. (2011). Synthesis and characterization of nanocrystallized In_2_S_3_ thin films via CBD technique. Mater. Chem. Phys..

[43] Saikat C., Kamakhya P.M., Arunava A., Aga S., Sukriti J., Nilanjan H., Ashok R., Babue P.D., Mukesh S., Anoop K.M. (2019). Dislocations and particle size governed band gap and ferromagnetic ordering in Ni doped ZnO nanoparticles synthesized via co-precipitation. Ceram. Int..

[44] Mustapha S., Ndamitso M.M., Abdulkareem A.S., Tijani J.O., Shuaib D.T., Mohammed A.K., Sumaila A. (2019). Comparative study of crystallite size using Williamson-Hall and Debye-Scherrer plots for ZnO nanoparticles. Adv. Nat. Sci. Nanosci. Nanotechnol..

[45] Imen B.E., Nejeh H., Amine M., Ridha A. (2020). Photoconduction, dielectric and photoluminescence properties of Cu^2+^: ZnO nanoparticles elaborated by a polyol method. Phase Transit..

[46] Li G., Wang H., Wang Q., Zhao Y., Wang Z., Du J., Ma Y. (2015). Structure and properties of Co-doped ZnO films prepared by thermal oxidization under a high magnetic field. Nanoscale Res. Lett..

[47] Li W., Wang G., Chen C., Liao J., Li Z. (2017). Enhanced visible light photocatalytic activity of ZnO nanowires doped with Mn^2+^ and Co^2+^ ions. Nanomaterials.

[48] Karmakar R., Neogi S.K., Banerjee A., Bandyopadhyay S. (2012). Structural, morphological, optical and magnetic properties of Mn doped ferromagnetic ZnO thin film. Appl. Surf. Sci..

[49] Tauc J., Grigorvici R., Yanca Y. (1966). Optical properties and electronic structure of amorphous germanium. Phys. Status Solidi.

[50] Pancove J. (1975). Optical Processes in Semiconductors.

[51] Kaur G., Mitra A., Yadav K.L. (2015). Pulsed laser deposited Al-doped ZnO thin films for optical applications. Prog. Nat. Sci. Mater. Int..

[52] Nafees M., Liaqut W., Ali S., Shafique M.A. (2013). Synthesis of ZnO/Al:ZnO nanomaterial: Structural and band gap variation in ZnO nanomaterial by Al doping. Appl. Nanosci..

[53] Ahmad M., Ahmed E., Zhang Y., Khalid N.R., Xu J., Ullah M. (2013). Preparation of highly efficient Al-doped ZnOphotocatalyst by combustion synthesis. Curr. Appl. Phys..

[54] Sudaresan A., Bhargavi R., Rangarajan N., Siddesh U., Rao C.N.R. (2006). Ferromagnetism as a universal feature of nanoparticles of the otherwise nonmagnetic oxides. Phys. Rev. B.

[55] Panigrahy B., Aslam M., Misra D.S., Ghosh M., Bahadur D. (2010). Defect-related emissions and magnetization properties of ZnO nanorods. Adv. Funct. Mater..

[56] Xing G., Wang D., Yi G., Yang L., Gao M., He M., Yang J., Ding J., Sum T.C., Wu T. (2010). Correlated d0 ferromagnetism and photoluminescence in undoped ZnO nanowires. Appl. Phys. Lett..

[57] Kapilashrami M., Xu J., Strom V., Rao K.V., Belova L. (2009). Transition from ferromagnetism to diamagnetism in undoped ZnO thin films. Appl. Phys. Lett..

[58] Vijayaprasath G., Murugan R., Mahalingam T., Ravi G. (2015). Comparative study of structural and magnetic properties of transition metal (Co, Ni) doped ZnO nanoparticles. J. Mater. Sci..

[59] Kaspar T.C., Droubay T., Heald S.M., Nachimuthu P., Wang C.M., Shutthanandan V., Johnson C.A., Gamelin D.R., Chambers C.A. (2008). Lack of ferromagnetism in *n*-type cobalt-doped ZnO epitaxial thin films. New J. Phys..

[60] Lawes G., Risbud A.S., Ramirez A.P., Seshadri R. (2005). Absence of ferromagnetism in Co and Mn substituted polycrystalline ZnO. Phys. Rev. B.

[61] Rao C.N.R., Deepak F.L. (2005). Absence of ferromagnetism in Mn-and Co-doped ZnO. J. Mater. Chem..

[62] Choi B.K., Chang D.H., Yoon Y.S., Kang S.J. (2006). Optical characterization of ZnO thin films deposited by Sol-gel method. J. Mater. Sci. Mater. Electron..

[63] Liu W., Li W., Hu Z., Tang Z., Tang X. (2011). Effect of oxygen defects on ferromagnetic of undoped ZnO. J. Appl. Phys..

[64] Chen Y., Goering E., Jeurgens L., Wang Z., Phillipp F., Baier J., Tietze T., Schutz G. (2013). Unexpected room-temperature ferromagnetism in bulk ZnO. Appl. Phys. Lett..

[65] Gacic M., Jakob G., Herbort C., Adrian H., Tietze T., Brück S., Goering E. (2007). Magnetism of Co-doped ZnO thin films. Phys. Rev. B.

